# Validity of a family-centered approach for assessing infants’ social-emotional wellbeing and their developmental context: a prospective cohort study

**DOI:** 10.1186/s12887-017-0898-5

**Published:** 2017-06-15

**Authors:** Margriet Hielkema, Andrea F. De Winter, Sijmen A. Reijneveld

**Affiliations:** Department of Health Sciences, University Medical Center Groningen, University of Groningen, Antonius Deusinglaan 1, 9713 AV Groningen, Groningen, The Netherlands

**Keywords:** Family-centered care, Well-child care, Social-emotional development, Risk identification

## Abstract

**Background:**

Family-centered care seems promising in preventive pediatrics, but evidence is lacking as to whether this type of care is also valid as a means to identify risks to infants’ social-emotional development. We aimed to examine the validity of such a family-centered approach.

**Methods:**

We conducted a prospective cohort study. During routine well-child visits (2–15 months), Preventive Child Healthcare (PCH) professionals used a family-centered approach, assessing domains as *parents’ competence, role of the partner, social support, barriers within the care-giving context,* and *child’s wellbeing* for 2976 children as protective, indistinct or a risk. If, based on the overall assessment (the families were labeled as “cases”, *N* = 87), an intervention was considered necessary, parents filled in validated questionnaires covering the aforementioned domains. These questionnaires served as gold standards. For each case, two controls, matched by child-age and gender, also filled in questionnaires (*N* = 172). We compared PCH professionals’ assessments with the parent-reported gold standards. Moreover, we evaluated which domain mostly contributed to the overall assessment.

**Results:**

Spearman’s rank correlation coefficients between PCH professionals’ assessments and gold standards were overall reasonable (Spearman’s rho 0.17–0.39) except for the domain *barriers within the care-giving context.* Scores on gold standards were significantly higher when PCH assessments were rated as “at risk” (overall and per domain).We found reasonable to excellent agreement regarding the absence of risk factors (negative agreement rate: 0.40–0.98), but lower agreement regarding the presence of risk factors (positive agreement rate: 0.00–0.67). An “at risk” assessment for the domain *Barriers or life events within the care-giving context* contributed most to being overall at risk, i.e. a case, odds ratio 100.1, 95%-confidence interval: 22.6 - infinity.

**Conclusion:**

Findings partially support the convergent validity of a family-centered approach in well-child care to assess infants’ social-emotional wellbeing and their developmental context. Agreement was reasonable to excellent regarding protective factors, but lower regarding risk factors.

**Trial registration:**

Netherlands Trialregister, NTR2681. Date of registration: 05–01-2011, URL: http://www.trialregister.nl/trialreg/admin/rctview.asp?TC=2681.

**Electronic supplementary material:**

The online version of this article (doi:10.1186/s12887-017-0898-5) contains supplementary material, which is available to authorized users.

## Background

A child’s development is influenced by the context in which it grows up, as well as by in addition to for example biological factors [1]. On the one hand, a positive and supportive context, as provided by adequate parenting, may optimize a child’s development -within the possibilities of its genetic and biological make-up- [2, 3]. On the other hand, a less favorable context, as with marital conflict, maternal depression, or poverty, may have a negative influence [4, 5]. The development of young children in particular is intertwined with their developmental context. The younger children are, the more they rely on their developmental context for the regulation of emotions and behavior [6].

Family-centered care may help to optimize a child’s developmental context and in turn the child’s social-emotional development [7], and has also been recognized as playing an important role in the quality of preventive pediatrics, as reflected by guidelines like Bright Futures of the American Academy of Pediatrics [8]. Table 1 presents the core principles of Family-centered care according to the American Academy of Pediatrics [9]. In the Netherlands, a family-centered approach, hereafter called the family-centered approach, has been introduced in Preventive Child Healthcare (PCH) with, among others, the mandatory task of monitoring children’s social-emotional development and their developmental context [10]. PCH, like well-child care in other countries, involves only preventive activities, and is offered free of charge to the total Dutch population. More than 90% of all families with children frequently visit PCH.

**Table 1:** Core principles of family-centered care according to the American Academy of Pediatrics

1. Respecting each child and his or her family
2. Honoring racial, ethnic, cultural, and socioeconomic diversity and its effect on the family’s experience and perception of care
3. Recognizing and building on the strengths of each child and family, even in difficult and challenging situations and respecting different methods of coping
4. Supporting and facilitating choice for the child and family about approaches to care and support
5. Ensuring flexibility in organizational policies, procedures, and provider practices so services can be tailored to the needs, beliefs, and cultural values of each child and family
6. Sharing honest and unbiased information with families on an ongoing basis and in ways they find useful and affirming
7. Providing and/or ensuring formal and informal support (eg, family-to-family support) for the child and parent(s) and/or guardian(s) during pregnancy, childbirth, infancy, childhood, adolescence, and young adulthood
8. Collaborating with families at all levels of health care, in the care of the individual child and in professional education, policy making, and program development
9. Empowering each child and family to discover their own strengths, build confidence, and make choices and decisions about their health

The newly implemented family-centered approach aims to build a trustful and supportive relationship with parents and to empower parenting skills, with the aim of enhancing children’s developmental context. Next to these more general relational and participatory principles, the family-centered approach incorporates a systematic component, reflected by the use of a checklist to identify risk and protective factors for infants’ social-emotional development [10]. Contents of the checklist are based on the bio-ecological model of Bronfenbrenner, which describes the factors that influence human development at different levels, taking into account both the child and its developmental context, and the interaction between the two [11]. In the family-centered approach, the bio-ecological model is reflected in the following domains related to children’s social-emotional wellbeing: *competence of the parent, role of the partner, social support, life events within the care giving context, and wellbeing of the child*. Using the information on all domains, PCH professionals draw an overall conclusion about the child’s social-emotional wellbeing.

The family-centered approach seems promising for preventive pediatrics. However, evidence is lacking as to whether this approach allows for valid assessment of protective and risk factors regarding infants’ social-emotional development in well-child care. Therefore, the aim of this study was to examine this validity, and to compare the agreement between PCH professional’s assessments and parents’ responses in validated questionnaires.

## Methods

The current study was part of a large quasi-experimental study comparing the family-centered approach with care-as-usual in Dutch PCH. For the current study, we used data only of participants fully offered the family-centered approach in order to make an adequate assessment of its performance. The study was approved by the Medical Ethics Committee of the University Medical Center Groningen. Below, we summarize its design; further details have been described in a separate design paper [12].

### Participants

We used data from a cohort of 2976 participants in the family-centered condition who gave written informed consent at the start of the study, when their child was about 2 months old. When they consented, parents were informed that they could be asked to participate in an extra interview when PCH professionals provided any extra care for the infants’ social-emotional development. Of the 2976 participants, 114 were asked by PCH professionals, i.e. nurses and medical doctors, to participate in such interviews because of the need for an additional activity regarding the child’s social-emotional development (e.g., an additional phone call, appointment or extra well-child visit to assess the situation more in depth, or an intervention like a referral to a child psychologist); 87 parents (76%) agreed on this. Three families were seen twice and two families three times, because more than once during the period from 2 to 18 months an additional activity from PCH was needed. For the analysis, we took into account only the first identification of each family. For all cases, two “control” families, matched by age and gender of the child, but for whom PCH performed no additional activity, were invited. Of 2 of the 174 controls, data could not be used because their medical records did not include data regarding the family-centered approach.

### Intervention and procedures

The family-centered approach is the only approach in Dutch PCH that takes into account the child within its context and can be used during all routine well-child visits from birth onwards. The family-centered approach strongly focuses on building rapport with parents. Where possible, PCH professionals attune their care to the needs and wishes of each family by taking the parents’ (or caregivers’) point of view as basis for the well-child visit and treating them as equal partners and experts on their child [13]. Through empowering communication, PCH professionals aim to enhance parents’ confidence and parenting skills, thereby trying to improve the child’s developmental context. Next to these more general principles, the approach consists of a checklist that covers five domains associated with children’s social-emotional development (see Additional file 1: Appendix 1 for the domains and questions regarding these domains) [10]. The questions for each domain form a guideline for PCH professionals for their conversation with parents. The professionals used the family-centered approach during each routine well-child visit for children aged 2, 3, 4, 6, 7,5, 9, 11, and 14 months. For each domain, PCH professionals registered information within the child’s medical record as *not discussed, protective, indistinct*, or at *risk*. The term *protective* reflected either a stable or enhancing situation for both high- and low-risk children, conform the use of promotive factors as previously described by Sameroff [14]; *indistinct* reflected a situation that could not correctly be labeled either as *protective* nor *at risk*. Subsequently an explanation in free text could be provided. Based on the appraisal of all the domains, the parent and the PCH professional jointly decided whether there were any causes for concern, and an overall conclusion was drawn as *fine*, *not optimal* or a *problem*. In cases of concern, an additional activity aimed at the social-emotional development of the child was planned, for example an additional appointment to assess the situation more in depth or an intervention like a referral to a child psychologist.

All PCH professionals attended 4 days of training before starting with the family-centered approach. Within one month after training they had to videotape two well-child visits in which they used the family-centered approach. The videos were discussed with trainers who used standardized guidelines to determine the adequacy of trainees’ performance [10]. This procedure was repeated until the performance of the family-centered approach was rated as adequate. Furthermore, the PCH professionals attended supervision every three months. Before our study started, we trained all these professionals for half a day, providing practical as well as theoretical information on the study as, for example, how to include participants and how to provide cases for the study.

All cases and controls were contacted by trained interviewers from the research institute for a questionnaire-based interview at the parents’ home (see Table 2 for all the questionnaires used), five families preferred filling in the questionnaire themselves and were mailed. Whenever feasible, appointments were made within one week after the routine well-child visit, this was possible for 53% of the interviews. In case of intervals longer than one week, we checked with PCH professionals about possible changes in the situation during the time between the well-child visit and the interview. Families participated in the interview only if no relevant changes had taken place since the last well-child visit.

**Table 2:** Parent-report questionnaires used as gold standards for the domains of the family-centered care approach

Domain of the Family-centered approach	Criterion	Nr. of items	Measuring	Information on reliability and validity (*and Cronbach’s alpha in our study*)	Cut-off scores	References
Wellbeing of the child	Ages and Stages Questionnaire Social Emotional (*ASQ-SE*) (versions 6, 12 and 18 months)	22–29	Social-emotional development of the child	Cronbach’s alpha 0.82. Test-retest reliability 0.94. Sensitivity 0.75–0.89. Specificity 0.82–0.96. *(0.41–0.69)*	High >2 sd	[27]
Competence of the parent	Dutch Parenting Stress Index (*PSI*) (4 subscales)	11	Parental competence and attachment	Cronbach’s alpha 0.92–0.96. Good construct and criterion validity* *(0.82)*	High >90th pct	[28]
	Parenting Tasks Checklist or Problem Setting and Behavior Checklist *(PSBC)(Setting Self-Efficacy subscale)*	14	Perceived ability of the primary caretaker in mastering problem situations	Cronbach’s alpha 0.91 *(0.89)*	Low <10th pct	[29]
	Parental Sense of Competence scale (*PSOC*)	16	Competence of the parent	Cronbach’s alpha 0.70–0.88. Test-retest reliability 0.46–0.82. Good construct validity. *(0.84)*	High: >2 sd	[30]
	SF-12 Health Survey *SF-12 mental* *SF-12 physical*	12	Health status (physical and mental) of the parent	Abbreviated version of the validated 36-Item Short Form Health Survey. Correlations betwee SF-36 and SF-12 are high, i.e.0.94–0.97 *(0.67–0.71)*	Low: <10th pct Low: <10th pct	[31]
Role of the partner	McMaster Family Assessment Device (*FAD*) (General Functioning subscale)	12	Emotional relationships within families	Cronbach’s alpha 0.66–0.81.Good construct validity. *(0.94)*	High: >90th pct	[32]
	Dutch Parental Stress Index (*PSI*) (subscale partner)	5	Having a child and its effect on the relationship between partners	Cronbach’s alpha 0.92–0.96. Good construct and criterion validity* *(0.71)*	High: >90th pct	[28]
Social support	Social Support List, short version (*SSL*) *Received* *Shortage*	12	Social support	Cronbach’s alpha 0.69–0.96, Construct and criterion validity sufficient* *(0.74–0.79)*	Low: <2 sd High: >90th pct	[33]
	Loneliness-score Social Emotional	11	Feelings of overall, emotional and social loneliness	Cronbach’s alpha 0.80–0.90. sufficient content validity. *(0.80–0.85)*	High: >90th pct High: >90th pct High: >90th pct	[34]
Perceived barriers or life events within the care giving context of the child	Questionnaire on the material or social deprivation of a child due to shortage of money (*deprivation questionnaire)*	15	The material or social deprivation of a child due to shortage of money	Cronbach’s alpha 0. 89. *(0.63)*	High: > 90th pct	[35]
	Dutch Parental Stress Index (*PSI*)(subscale life events)	17	Life events happened in the past year	Cronbach’s alpha 0.92–0.96. Good construct and criterion validity*	High: >2 sd	[28]

*Sd: standard deviation*

*Pct: percentile*

### Measures

PCH professionals assessed all five domains of the family-centered approach by using the questions in the checklist (see Additional file 1: Appendix). They evaluated information on these domains as *not discussed, protective, indistinct*, or at *risk* and subsequently rated the overall situation as *fine, not optimal* or *a problem*, as described under the heading of “Procedures”. By means of an interview, parents filled out questionnaires with good construct and/or criterion validity. These questionnaires served as gold standard for the domains of the family-centered approach. The questionnaires are shown in Table 2.

If for controls specific ratings for domains or the overall conclusion were missing, those from the subsequent visit were used. This was done only when that rating contained a note stating that nothing had changed since the previous visit*.* Furthermore, in the case of missing ratings on domains for both controls and cases, we coded domains as *protective* if free text explicitly stated that everything was fine and as *indistinct* when free text stated that problems or barriers existed*.* For 44 controls and 15 cases we coded one or more domains as so described.

Moreover, we assessed the following background characteristics of parents: *age, educational level, working participation, country of birth* and furthermore the *family composition*, and having *one or more children.* We used this information from the child’s medical record or, if records lacked data on this, from the parent reported questionnaire at the start of our study*.* Educational level reflected the highest obtained level for one of both parents and was divided into low (primary school or less, lower vocational or lower general secondary education), medium (intermediate vocational education, intermediate or higher secondary education) and high (higher vocational education or university).

### Analysis

Analyses were performed using the Statistical Package for Social Sciences (SPSS) version 20. The statistical significance level was set at.05. We first compared background characteristics of cases and controls by using Chi-square tests or Fisher’s exact tests in case of more than 20% of cells with an expected count <5.

Second, we assessed the convergent validity by computing Spearman’s rank correlation coefficients between PCH professionals’ assessments (*protective, indistinct* or *at risk*) and the gold standards for the domains of the family-centered approach. Correlation coefficients >.30 were interpreted as reasonable [15]. Additionally, we compared scores on the gold standards for cases versus controls, i.e. PCH-initiated intervention versus no intervention, and per domain (assessed as at risk versus assessed as not at risk) using conditional logistic regression analysis to take into account the matching by age and gender [16]. Effect sizes were then computed [16], effect sizes from 0.10–0.30 were interpreted as small, 0.30–0.50 as medium and >0.50 as large [17].

Third, we assessed the agreement between PCH professionals’ assessments and the gold standards regarding the domains of the family-centered approach. We calculated percentages of agreement overall, and for cases and controls separately using the mean of (P(PCH professional’s assessment *risk*/ gold standard *risk*) + P(PCH professional’s assessment *protective*/ gold standard *protective*)). Furthermore, for a better understanding of our results, we calculated both the positive agreement (Ppos), i.e. the agreement regarding the presence of risk factors, and negative agreement (Pneg), i.e. the agreement on the absence of risk factors [18]. For this purpose we dichotomized the scores of PCH professionals’ assessments as *protective* versus *indistinct* or at *risk* per domain, and divided questionnaire scores into low and high scores. We based this latter dichotomization on the scores of controls; high scores were defined as more than two standard deviations higher than the mean, or, in case of skewed data, as higher than the 90th percentile. Whenever norm scores were available for a questionnaire, we also used these to dichotomize our data based.

Finally, we assessed which domains contributed most to PCH professionals’ overall assessments by calculating the percentages of risk assessments per domain for both cases and controls and performing conditional univariate logistic regression analysis to show to what extent each domain separately contributed to the overall conclusion of the PCH professional as to whether or not a child was at risk.

## Results

Background characteristics of both cases and controls are presented in Table 3. Regarding cases, mothers were more often below 20 years or over 40 years of age. Moreover, cases more often came from a one-parent household.

**Table 3:** Background characteristics of participants

	Cases (*N* = 87)	Controls (*N* = 172)	Total cohort^b^ (*N* = 2835)	*P*-value cases-controls^ϕ^/cases-total cohort
Gender				
Male	46 (52.9%)	90 (52.3%)	1420 (50.1%)	
Female	41 (47.1%)	82 (47.7%)	1414 (49.9%)	.61
Highest educational level of either parents				
Lower	4 (4.8%)	4 (2.4%)	119 (4.7%)	.06
Secondary	44 (57.9%)	77 (45.6%)	1099 (43.0%)	.03
Higher	28 (36.8%)	88 (52.1%)	1336 (52.3%)	
Parental age				
Mother				
Younger than 20	2 (2.3%)	1 (0.6%)	15 (0.6%)	.04^a^
20–40	81 (93.1%)	169 (98.8%)	2351 (96.6%)	.05^a^
40 years and over	4 (4.6%)	1 (0.6%)	59 (2.4%)	
Father				
Younger than 20	1 (1.2%)	1 (0.6%)	5 (0.2%)	.73^a^
20–40	70 (81.4%)	141 (84.9%)	2092 (89.6%)	.03
40 years and over	15 (17.4%)	24 (14.5%)	239 (10.2%)	
Employment status parent				
One of both or both parents have	85 (97.7%)	167 (97.7%)	1206 (94.4%)	1.00^a^
paid work				.23^a^
None of both parents has paid	2 (2.3%)	4 (2.3%)	72 (5.6%)	
Work				
Country of birth parent				
One or both born in the Netherlands	86 (98.9%)	169 (100.0%)	2460 (99.3%)	.34^a^
Both born outside the Netherlands	1 (1.1%)	0 (0.0%)	86 (0.7%)	.48^a^
Family composition				
Two parents household	78 (91.1%)	171 (99.4%)	2046 (96.9%)	.01^a^
One parent household	7 (8.2%)	1 (0.6%)	65 (3.1%)	.05^a^
Number of children				
First child	37 (43.4%)	81 (47.1%)	1215 (42.9%)	.59
More children	48 (56.5%)	91 (52.9%)	1620 (55.3%)	1.00

^a^based on Fisher’s exact test

^b^participants for whom data was available, cases excluded

^ϕ^for gender the *p*-value was not given for the comparison between cases and controls because of the matching by gender

### Convergent validity

Table 4 shows Spearman’s rank correlations between domains rated as *protective* versus *indistinct* or *at risk* and scores on the related questionnaires. All correlations were statistically significant (ranging from .17 to .39 with around two third >.30) and highest for the domains that the questionnaire should cover, except for the PSBC, the Loneliness score Emotional and the Deprivation Questionnaire.

**Table 4:** Comparison of scores on parent-reported questionnaires (i.e. gold standards) between cases and controls

	Cases (intervention based on overall assessment)			Controls (no intervention based on overall assessment)					
	*N*	Mean	(sd)	*N*	Mean	(sd)	*P*-value	Effect size Cohen’s d	Spearman’s rho
Wellbeing of the child									
ASQ-SE	84	0.41^a^	(1.1)	165	−0.21^a^	(.84)	<.001	.33	.306***
Competence of the parent									
PSI	86	23.4	(8.9)	169	18.3	(5.4)	<.001	.06	.322***
PSOC	85	36.6	(10.9)	167	30.2	(7.2)	<.001	.30	.269***
PSBC^b^	87	8.8	(1.0)	172	9.2	(0.8)	.001	.04	−.208***^c^
SF-12 mental^b^	87	44.2	(11.1)	172	52.9	(7.8)	<.001	.05	−.371***
SF-12 physical^b^	87	49.5	(8.6)	172	50.5	(8.2)	.45	.01	−.169***
Partner									
FAD	82	21.2	(10.0)	167	15.3	(3.6)	<.001	.10	.394***
PSI (partner)	79	9.6	(3.2)	172	7.7	(2.4)	<.001	.16	.269***
Social support									
SSL received^b^	87	15.2	(3.1)	172	15.7	(2.8)	.17	.03	−.231***
SSL shortage	86	8.4	(3.1)	172	6.8	(1.3)	<.001	.23	.375***
Loneliness score	87	2.7	(3.0)	172	1.1	(1.9)	<.001	.17	.293***
Social	87	1.1	(1.5)	172	0.6	(1.0)	.002	.20	.375***
Emotional	87	1.6	(1.8)	172	0.5	(1.2)	<.001	.28	.394***^c^
Barriers or life events within care-giving context									
Deprivation Questionnaire	86	0.5	(1.4)	171	0.1	(0.3)	.004	.49	.272***^c^
PSI (life events)	87	1.5	(1.0)	172	1.3	(1.0)	.08	.13	.212**

^a^Based on Z-scores

^b^Lower scores reflect worse outcomes

^c^Spearman’s rho was higher between the questionnaire scores and one of the other domains than with the intended corresponding domain

**p-value < .05

***p-value <.01

Scores on the parent-reported questionnaires were mostly higher for children for whom PCH professionals initiated an intervention (cases) than for children for whom they did not so (controls); see mean scores in Table 4. Effect sizes ranged from marginal to medium. We found similar effect sizes for the PCH professionals’ conclusions per domain *protective* versus *indistinct* or *at risk*.

### Agreement between PCH professionals and parents per domain

Table 5 shows findings regarding agreement between PCH professionals and parents per domain, for cases and controls separately and combined. We found reasonable to excellent levels of agreement (61%–98%). Overall we found higher agreement for cases than for controls, especially for the domains *Social support* and *Perceived barriers or life events within the care giving context* (agreement between 63%–85% versus 46%–59% for cases and controls respectively). For the domain *Wellbeing of the child*, the agreement for controls was higher than for cases (98% versus 67%). The agreement on the absence of risk factors (Pneg), which in this study indicated the presence of protective factors (see “intervention and procedures”), was overall satisfactory, and was especially high for controls. The agreement on the presence of risk factors (Ppos) was low (lowest for controls). For cases, PCH professionals frequently identified a *risk* where parents scored low on the accompanying questionnaires whereas the discrepancy ‘professional: *protective*’; ‘parent: *risk*’ occurred more frequently among controls.

**Table 5:** Agreement between assessments of PCH professionals and scores on parent-reported gold standards per domain

PCH-professional/parent		risk^a^/risk	risk^a^/protective	protective/risk	protective/protective			
	N					Agreement	Ppos	Pneg
Wellbeing of the child								
ASQ-SE	236	5	41	1	189	83%	.19	.90
Cases/Controls	82/154	4/1	36/5	1/0	41/ 148	67%/98%	.18/.29	.69/.98
Competence of the parent								
PSI	250	22	32	21	175	68%	.45	.87
Cases/Controls	84/166	19/3	29/3	9/12	27/ 148	58%/59%	.50/.29	.59/.95
PSOC	247	14	41	10	182	70%	.35	.88
Cases/Controls	83/164	14/0	35/6	5/5	29/ 153	59%/48%	.41/.00	.59/.96
PSBC	254	14	41	21	178	61%	.31	.85
Cases/ Controls	85/169	13/1	36/5	7/14	29/ 149	55%/52%	.38/.10	.57/.94
SF-12 mental	254	23	32	22	177	68%	.46	.87
Cases/Controls	85/169	19/4	30/2	10/12	26/ 151	56%/62%	.49/.36	.57/.96
SF-12 physical	254	8	47	18	181	55%	.20	.85
Cases/Controls	85/169	7/1	42/5	2/16	34/ 147	61%/51%	.24/.09	.61/.93
Role of the partner								
FAD	204	23	14	22	145	71%	.56	.89
Cases/Controls	72/132	23/0	12/2	11/11	26/ 119	68%/49%	.67/.00	.69/.95
PSI (partner)	206	18	16	27	145	63%	.46	.87
Cases/Controls	69/137	15/1	17/1	14/13	23/ 122	55%/53%	.49/.13	.60/.95
Social support								
SSL received	205	4	24	0	177	94%	.25	.94
Cases/Controls	71/134	4/0	20/4	0/0	47/ 130	85%/49%	29./.00	.82/.98
SSL shortage	205	15	13	20	157	65%	.48	.90
Cases/Controls	71/134	13/2	11/2	11/9	36/ 121	65%/58%	.54/.27	.77/.96
Loneliness score	205	14	14	13	164	72%	.51	.92
Cases/Controls	71/134	12/2	12/2	5/8	42/ 122	74%/59%	.59/.29	.83/.96
Social	205	10	18	9	168	71%	.43	.93
Cases/Controls	71/134	9/1	15/3	4/5	43/ 125	72%/57%	.49/.20	.82/.97
Emotional	205	14	14	16	161	69%	.48	.91
Cases/Controls	71/134	12/2	12/2	7/9	40/ 121	70%/58%	.56/.27	.81/.96
Perceived barriers or life events within the care giving context								
Deprivation questionnaire	202	11	47	7	137	68%	.29	.83
Cases/Controls	63/139	11/0	37/10	0/7	15/ 122	64%/46%	.37/.00	.45/.93
PSI (life events)	203	3	55	3	142	61%	.09	.83
Cases/Controls	63/140	3/0	45/10	0/3	15/ 127	63%/46%	.12/.00	.40/.95

^a^Consists of domains assessed as a risk or indistinct

PCH: Preventive Child Healthcare

Ppos: positive agreement (on the presence of risk factors)

Pneg: negative agreement (on the absence of risk factors, in this study indicating the presence of protective factors)

### Contribution of domains to the PCH professional’s overall assessmen

Table 6 shows the rates of *at risk* and *protective* factors per domain that PCH professionals assessed, for cases versus controls, and the results of the univariate logistic regression analyses. The domain *Barriers or life events within the care-giving context* contributed the most to the overall assessment; if this domain was assessed as *at risk*, participants had an odds of about 100 to be assessed as a case, compared to when this domain was assessed as *protective*. Furthermore, when participants had two or more risk factors, they had a higher odds of being assessed as a case (odds ratio: 79.8; 95% confidence interval: 27.0–236.3).

**Table 6:** Contribution of domains to the overall assessment of the child by the PCH

	Cases (intervention based on overall assessment)	Controls (no intervention based on overall assessment)	OR (95% CI)
Wellbeing of the child			
Risk or indistinct	42 (49.4%)	6 (3.7%)	26.0 (8.1–84.2)
Protective	43 (50.6%)	155 (96.3%)	
Competence of the parent			
Risk or indistinct	49 (57.6%)	6 (3.6%)	22.8 (8.2–63.3)
Protective	36 (42.4%)	163 (96.4%)	
Role of the partner			
Risk or indistinct	40 (51.9%)	2 (1.5%)	61.7 (8.5–450.6)
Protective	37 (48.1.%)	135 (98.5%)	
Social support			
Risk or indistinct	24 (33.8%)	4 (3.0%)	19.9 (4.7–84.8)
Protective	47 (66.2%)	130 (97.0%)	
Barriers or life events within the care giving context			
Risk or indistinct	48 (76.2%)	10 (7.1%)	101.1 (22.6- infinity)
Protective	15 (23.8%)	130 (92.9%)	

Professional: results of conditional logistic regression analyses

*OR* odds ratio

*CI* confidence interval

## Discussion

In this study we examined the validity of a family-centered approach in well-child care for the early identification of concerns regarding infants’ social-emotional development. Results showed that PCH professionals’ assessments of infants’ social-emotional wellbeing and their developmental context, based on a family-centered approach, were associated with scores on gold standards. The agreement between PCH and parents per domain was overall satisfactory to excellent for protective factors, but not for risk factors. The domain *Barriers or life events within the care-giving context* contributed most to the PCH professional’s overall assessment of being at risk.

Our study was the first to assess extensively the validity of a family-centered approach, and our findings partially support its validity. These findings correspond with previous ones on the validity of this specific approach [10], and with findings on a similar approach, the Structured Problem Analysis of Raising Kids (SPARK), which also showed only partial support for the validity [19]. However, as our study covered more areas than only child development, family stress and family needs, it is difficult to make a comprehensive comparison of all findings.

We found that the agreement on protective factors was satisfactory to very good, especially for controls, but this was not always the case with risk factors. This finding suggests that the family-centered approach does not enable PCH professionals fully to assess risk factors. This is in line with previous findings of suboptimal identification by PCH regarding risk factors such as child abuse and psychosocial problems [20, 21]. Reasons for a suboptimal identification of risk factors could be the limited amount of time during well-child visits [22], or insufficient training to detect social-emotional problems. Moreover, identification of social-emotional problems in infants may also be more difficult [23].

Alternatively, the lower agreement regarding risk factors compared to protective factors may also reflect daily practice. First, with regard to cases, PCH professionals frequently assessed risk factors, whereas parents did not (yet). This may be the result of the preventive task of PCH and the family-centered approach, i.e. aiming to identify risks at an early stage to prevent (worsening of) problems whenever possible. The focus on risk factors may, however, entail the risk of stigmatization, and might interfere with the parental empowering advocated in the family-centered approach [10].

Second, PCH professionals also registered protective factors in some instances where parents scored high on the accompanying questionnaires, especially for controls. This may be because professionals take into account both protective and risk factors and are aware that protective factors can counterbalance risk factors. On the other hand, it may also be that professionals are reluctant to discuss certain topics with parents and tend to rate domains as protective, or that parents may be reluctant to discuss their worries or problems with PCH professionals. This issue evidently requires further study. If reluctance of parents to discuss is the issue, then more intense training in communication skills and more continuity of PCH professionals might contribute to parents’ disclosure [24].

The domain *Barriers or life events within the care-giving context* contributed the most to the PCH professionals’ overall assessment of being at risk. This corresponds with findings that, for example, poverty can be a risk for children’s social-emotional development [5]. However, studies also show that not the type of risk factor, but the number of risk factors is most predictive for the outcome, e.g. regarding child behavior [25]. This fits with our findings, since we found that whenever for participants two or more risk factors were assessed, they were more likely to be rated as a case.

### Strengths and limitations

Strengths of our study are its high response rates and its embedding in routine care. Since more than 90% of all families with children are visiting PCH services, and participants did not differ greatly from parents who did not participate in our study, chances are high that a majority of the at-risk families was included as well. Moreover, to optimize the coverage of all domains of the family-centered approach, we used a number of well evaluated questionnaires.

Some limitations of our study should, however, be discussed. First, no perfect ‘gold standards’ were available for the domains of the family-centered approach, a fact which may decrease the validity as measured. Though the questionnaires provide a valuable representation of the domains of the family-centered approach, some questionnaires covered only certain aspects of a domain. Unfortunately, comparing specific questionnaires with specific questions taken from the family-centered approach was not feasible because of a lack of data on some questions.

Second, in this study, we looked only at the contents of the family-centered approach, i.e. the checklist with questions as mentioned in the Additional file 1: Appendix. That fits with a starting point of family-centered care that the family is the constant in the child’s life. However, family-centered care is broader. Its relational component and participatory practices are of similar importance, as presented in Table 1 and in our description of the family-centered approach. In future research it would be interesting to assess what kinds of relational and participatory aspects of family-centered care are most essential to the identification of risk and protective factors, preferably including a stronger golden standard to define these aspects.

Third, we based our findings on single parent-reported questionnaires instead of using multi-informant and multi-method assessments. Fourth, we had to deal with missing values, although we imputed these in line with the principles of the family-centered approach.

## Conclusions

Our findings partially support the validity of a family-centered approach in well-child care. The family-centered approach seems particularly useful to assess protective factors, but less useful for evaluating risk factors for infants’ social-emotional development. For daily practice, one value of the family-centered approach lies in its assessment of protective factors, since building on strengths is recognized as important in optimizing children’s wellbeing [26]. It is a systematic approach that could and should allow for individualized care. The family-centered approach seems promising to support the development of young children.

## Additional file

Additional file 1: Appendix 1 Overview of the contents of the family-centered approach; the five domains and corresponding questions. Appendix 1 contains an overview of the five domains of the family-centered approach and its corresponding questions. (DOCX 13 kb)

## Abbreviation

PCH: Preventive Child Healthcare
