# Supplementary material for: Validity of a family-centered approach for assessing infants’ social-emotional wellbeing and their developmental context: a prospective cohort study
Source: BMC Pediatr. 2017 Jun 15;17:148. doi: 10.1186/s12887-017-0898-5 (PMC5472874; doi:10.1186/s12887-017-0898-5)
Supplement: Additional file 1: — Appendix 1 Overview of the contents of the family-centered approach; the five domains and corresponding questions. Appendix 1 contains an overview of the five domains of the family-centered approach and its corresponding questions. (DOCX 13 kb) [file 12887_2017_898_MOESM1_ESM.docx]

**Appendix 1:** Overview of the contents of the family-centered approach; the five domains and corresponding questions

**1. Competence of the primary caretaker**

- How do you like being a mother (of … children)?

- Does the situation correspond to what you expected?

- Do you feel uncertain or do you have any difficulties with certain aspects of care? If you have, what kind of aspects are these?

- To what extent do you have time for yourself or for other activities?

- How do you think your health is?

*Summarizing: the competence of the parent can be concluded as…*

**2. Role of the partner**

- How does your partner feel about having a child?

- To what extent does your partner contribute to the care of your child?

- To what extent are you satisfied with the contribution of your partner?

- To what extent do you and your partner agree on how to raise and care for children?

- What happens if you and your partner do not agree (about how to raise and care for children)?

- How is the relationship between you and your partner in general?

(in case of no relationship: how do you feel about that?)

- What is the impact of having a child on your relationship?

*Summarizing: the role of the partner can be concluded as…*

**3. Social support**

- Who supports you emotionally in caring for your child?

- Who supports you in practical terms in caring for your child?

- Who advises you about caring for your child?

- To what extent do you manage with the support you receive?

- Are you familiar with ways to enlarge your social network?

- To what extent are you in need of contact with other mothers with babies?

- How would you define your relationship with your own parents?

*Summarizing: the social support can be concluded as…*

**4. Perceived barriers or life events within the care-giving context of the child**

- Have there been any life events the past year?

If so: To what extent does this influence your contact with (name of the child)?

- How does the combination of work and child care services work for you?

- How is your financial situation?

- How is your housing situation?

- Are there any other circumstances that impact on your family?

*Summarizing: the perceived barriers or life events within the care giving context can be concluded as…*

**5. Wellbeing of the child**

- How is (name of the child) doing overall?

- How is (name of the child) developing on a social-emotional level according to you?

- How familiar are you with (name of the child)?

- How does (name of the child) respond to his/her environment?

- To what extent do you recognize different ways of crying?

*Summarizing: the wellbeing of the child can be concluded as…*
